# Supplementary figures and images for: RecPD: A Recombination-aware measure of phylogenetic diversity
Source: PLoS Comput Biol. 2022 Feb 22;18(2):e1009899. doi: 10.1371/journal.pcbi.1009899 (PMC8896707; doi:10.1371/journal.pcbi.1009899)

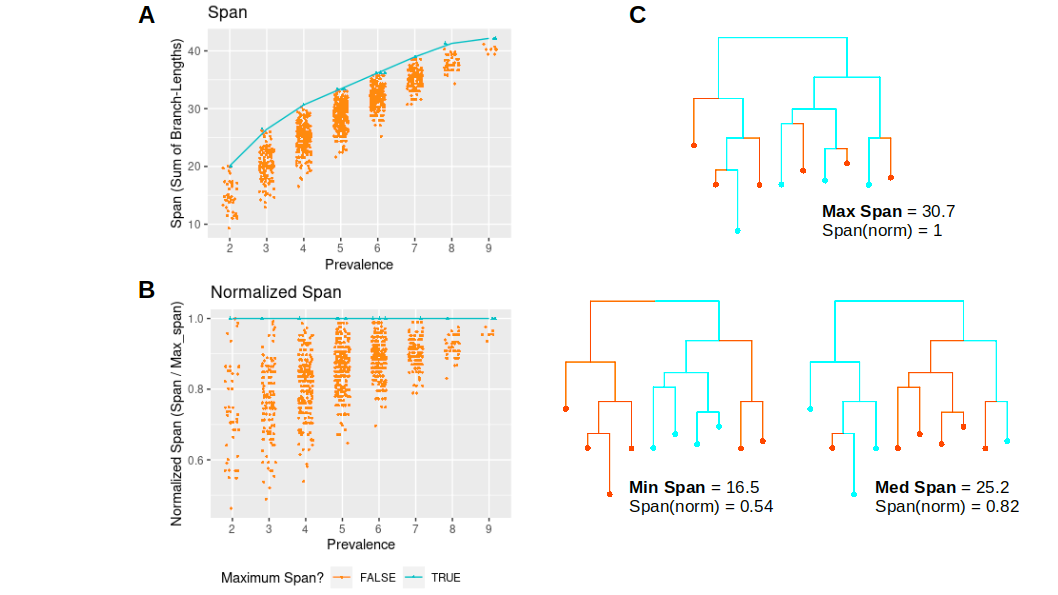

Supplement: S1 Fig — (A) Span is calculated by summing of branch-lengths joining tips in the phylogenetic tree possessing a gene family with a given level of prevalence. (B) Normalizing by the maximum possible sum of branch-lengths found at the same level of prevalence. (C) Example gene family distributions of prevalence = 4 mapped onto a tree of 10 tips, with maximum, minimum and median span values. (TIF) [file pcbi.1009899.s001.tif]

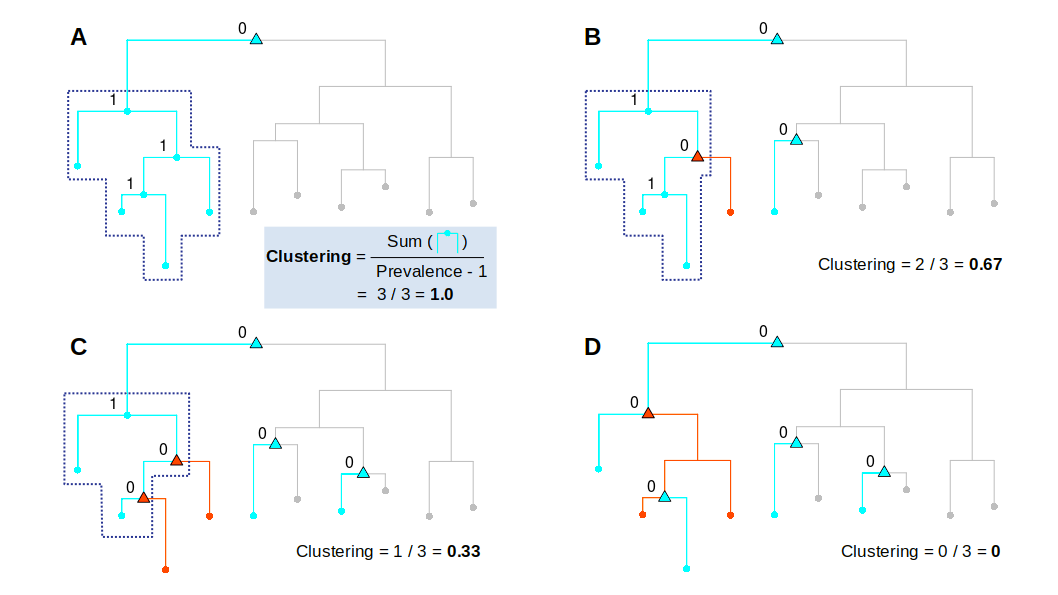

Supplement: S2 Fig — Clustering is calculated from the sum of the number of internal presence state nodes identified, normalized by the maximum clustering possible based on tip prevalence. (TIF) [file pcbi.1009899.s002.tif]

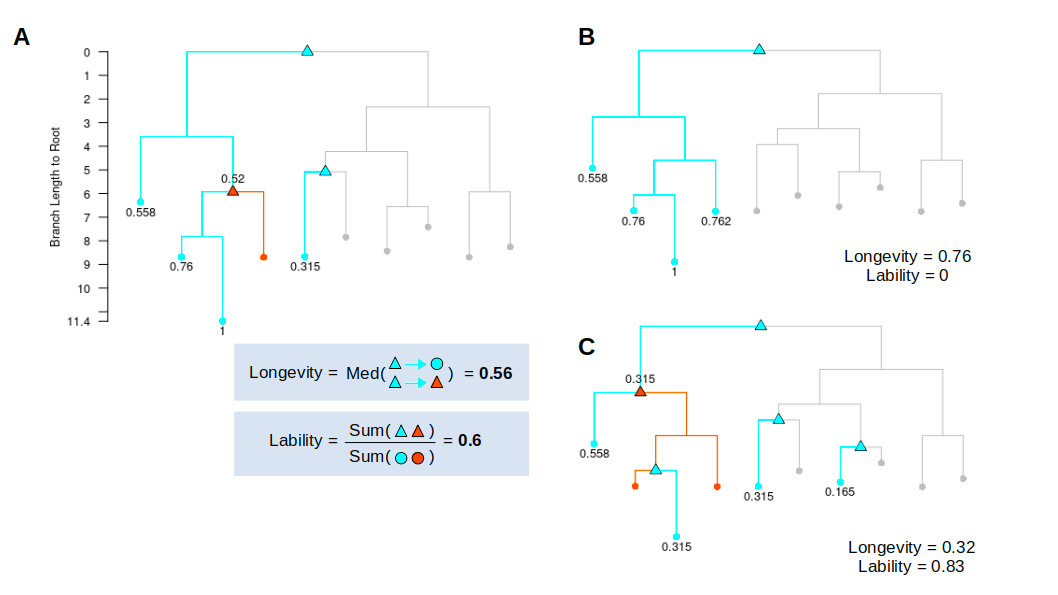

Supplement: S3 Fig — Longevity is calculated as the median branch-lengths of ancestral gain to loss internal nodes and presence state tips, normalized by the maximum root-to-tip distance of the phylogenetic tree. Lability is the corresponding sum of ancestral gain and loss nodes identified for each RecPD reconstructed gene-family lineage divided by the total number of gained and lost tips. Panels A–C show gene-family distributions of prevalence = 4 mapped onto a tree of 10 tips having approximately equal Longevity and Lability (A), High Longevity and low Lability (B) and low Longevity and high Lability (C). (TIF) [file pcbi.1009899.s003.tif]

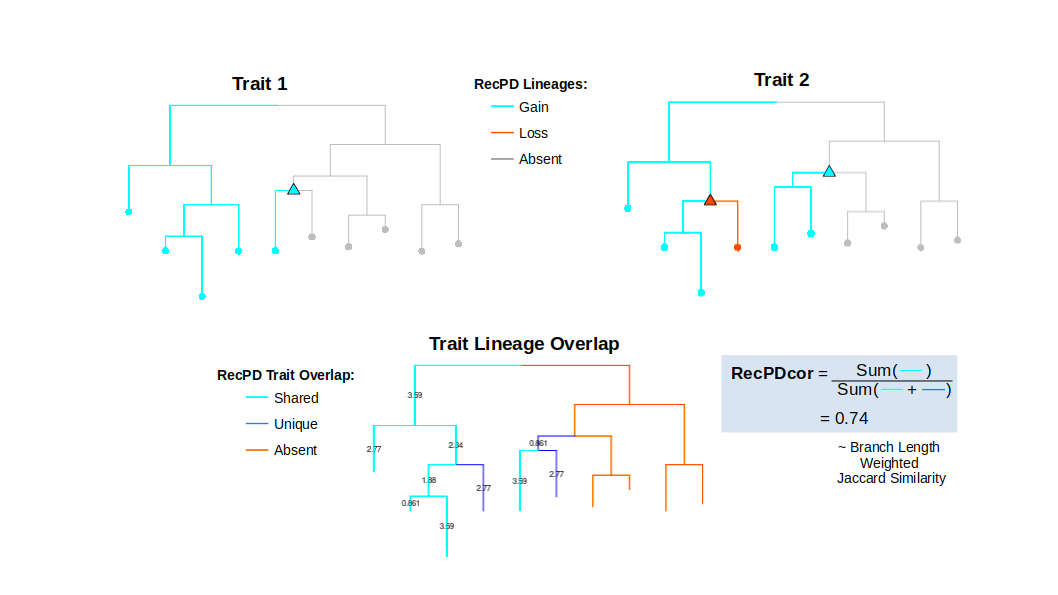

Supplement: S4 Fig — A pair of RecPD gene family reconstructions are merged into a consolidated phylogenetic tree with mutually present (teal), unique (blue), and mutually absent (red) ancestral branches identified. RecPDcor is then calculated as the sum of branch-lengths mutually present branches divided by the total sum of mutually present and unique branches. (TIF) [file pcbi.1009899.s004.tif]

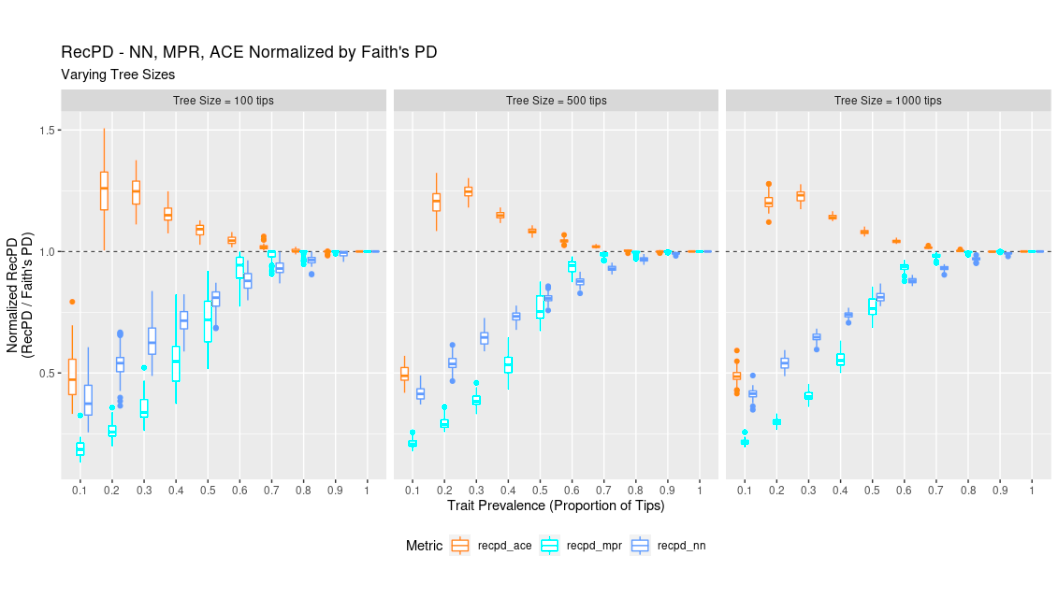

Supplement: S5 Fig — 50 random gene-family distributions were generated at each level of prevalence indicated, resulting to 451 distributions in total for each tree. (TIF) [file pcbi.1009899.s005.tif]

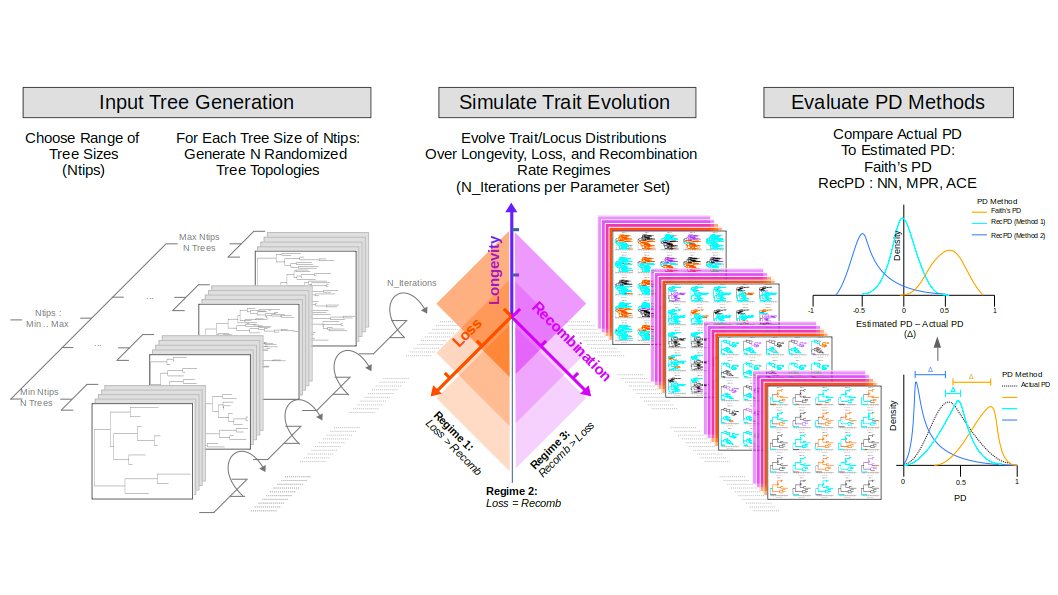

Supplement: S6 Fig — (TIF) [file pcbi.1009899.s006.tif]

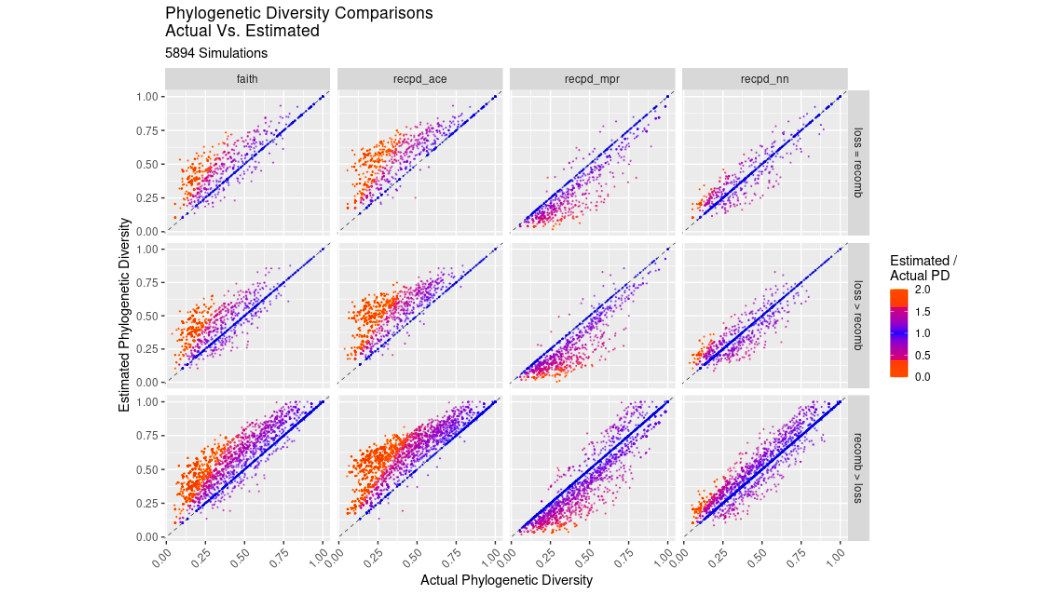

Supplement: S7 Fig — (TIF) [file pcbi.1009899.s007.tif]

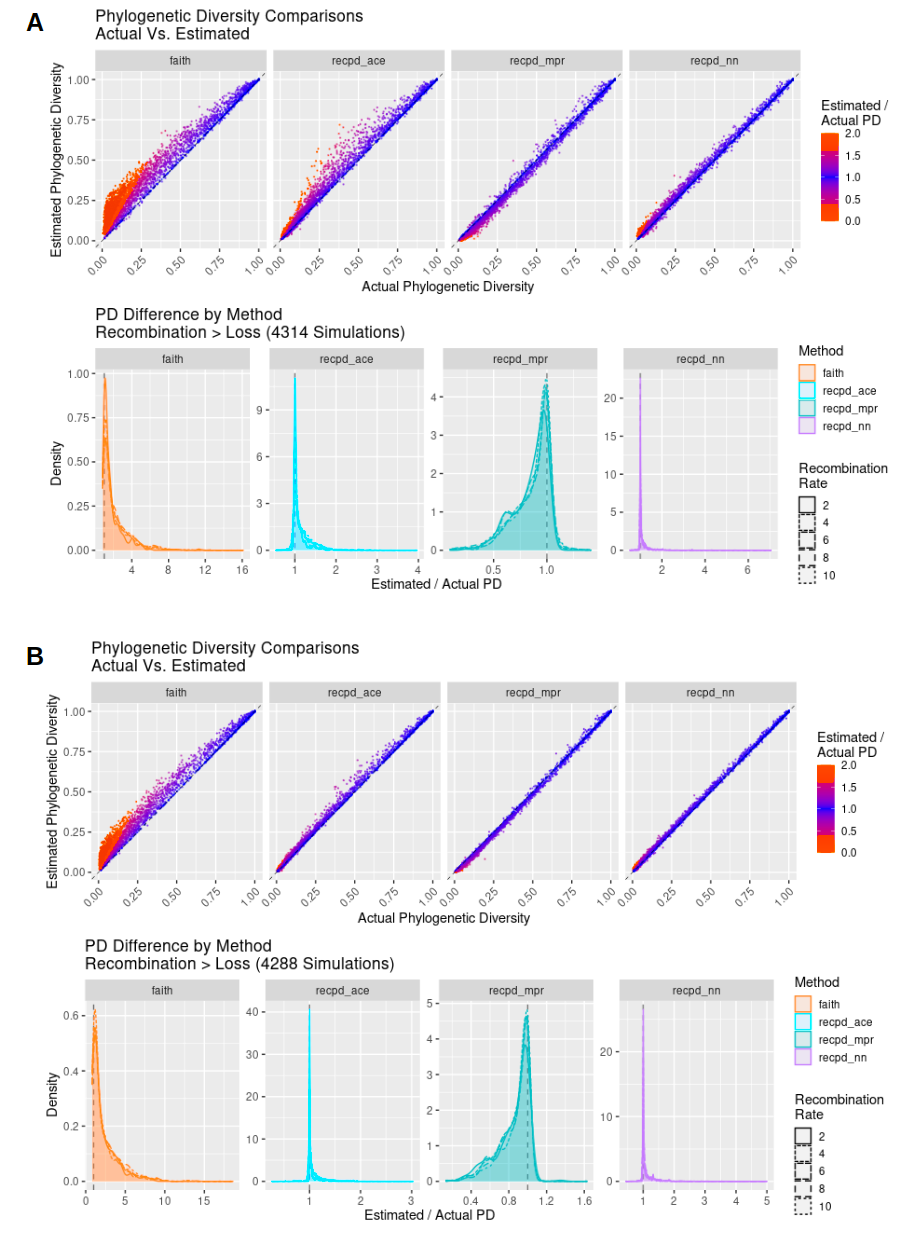

Supplement: S8 Fig — Difference of NN, MPR, and ACE and Faith’s PD compared to actual PD for evolved gene family distributions by rate regime. (A) Trees with 50 tips. (B) Trees with 100 tips. (TIF) [file pcbi.1009899.s008.tif]

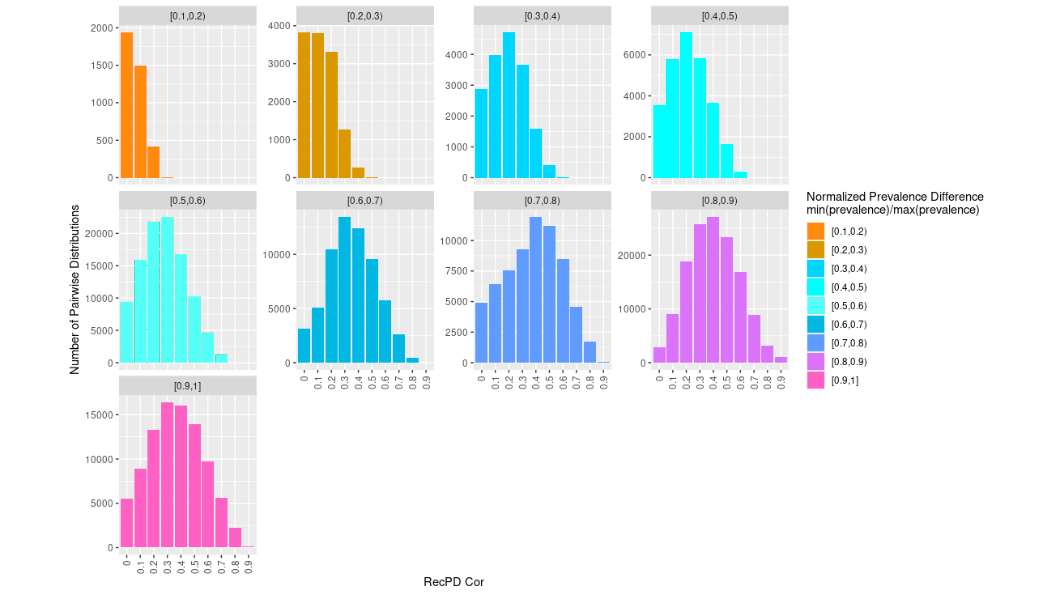

Supplement: S9 Fig — Facets represent the distribution of RecPDcor values binned by the normalized prevalence differences, min (prevalence) / max(prevalence), of each pairwise randomized gene-family distribution comparison compared. Note, normalized prevalence difference = 1 indicate distributions with identical prevalence. Results correspond to a test-case of all possible 1022 gene-family distributions mapped onto a tree of 10 tips. (TIF) [file pcbi.1009899.s009.tif]
