# Supplementary material for: RecPD: A Recombination-aware measure of phylogenetic diversity
Source: PLoS Comput Biol. 2022 Feb 22;18(2):e1009899. doi: 10.1371/journal.pcbi.1009899 (PMC8896707; doi:10.1371/journal.pcbi.1009899)
Supplement: Attachment — Submitted filename: RecPD Coverletter & Response to Reviewers - 2022.01.06.pdf [file pcbi.1009899.s013.pdf]

10 January 2022

RE: PCOMPBIOL-D-21-01774

Dear PLoS Computational Biology Editors,

Thank you very much for the opportunity to submit a revised manuscript. The reviewer comments were very helpful, and we feel that they have greatly improve the work. We have made all requested changes and provide details in the point-by-point list below.

Thank you once again for your consideration.

Sincerely,

David S. Guttman Ph.D.
Professor, Department of Cell & Systems Biology
Director, Centre for the Analysis of Genome Evolution & Function
University of Toronto
Toronto, Ontario, M5S3B2
Canada
david.guttman@utoronto.ca

REVIEWER #1

The authors present RecPD, a family of phylogenetic diversity measures that account for putative recombination and horizontal gene transfer events. In my opinion, this is topical work on a relevant topic, with a simple and elegant underlying idea. This is also one of the best written and most accessible manuscripts I've reviewed for a while – congratulations on this beautiful work!

The theoretical arguments and simulations for benchmarking seem reasonable and are well laid-out (with the caveats discussed below). The analysis of real data on P. syringae is very appropriate as an example use case for potential users.

That said, I have a few comments that I feel should be addressed prior to publication.

RESPONSE: Thank you very much for your positive and constructive comments.

While the writing is overall very clear and easy to follow, the terminology and language around "diversity" in the ecological sense is not always accurate. Local ('alpha') and between-community ('beta') diversities are discussed in the Introduction section, but there are no clear definitions of either, and it may be difficult for non-expert readers to place the RecPD family of measures among existing indices (beyond the comparison to Faith's PD).

Moreover, the authors introduce RecPD as "ecological" diversity measure, but the discussed use cases (both on simulated and real data) are not designed that way: rather than comparing samples of entire communities (e.g. of closely related strains), the authors compare lineage genomes (or traits) directly, as would be done in a comparative genomics study, basically using PD and RecPD as summary statistics on the trait. The authors address this by introducing RecPD as a measure on “features” that can be many things, but it remains unclear how RecPD would be used as an “ecological” diversity measure in the stricter sense in practice.

RESPONSE: The reviewer is correct that we were not as careful in our description of diversity metrics as we should have been. While many of the metrics we described in the introduction are most widely used in ecology, they are by no means limited to these applications. We have expanded our definitions of alpha and beta diversity and removed reference to ecology to better focus on their more general applicability. We hope that the new structure will be clearer to readers not familiar with the field.

Admittedly, while the P. syringae results give a good general example of RecPD’s usefulness, the manuscript does not leave me with a clear idea of how RecPD would be used in practice (and where it would not be appropriate). I believe that the authors should at least outline possible limitations with regards to data types and requirements: can RecPD handle imperfect phylogenies or missing data? How does the method scale computationally to larger problems?

RESPONSE: We have provided a second example to illustrate the application of RecPD in the case of growth phenotypes of the *Pseudomonas* genera. We hope breadth and contrast of these two examples of 1) effector families across strains, and 2) phenotypic diversity across species, will give readers a better appreciation for the potential utility of RecPD. We also agree with the reviewer that these analyses will face certain limitations, particularly regarding availability and quality of available datasets, and have incorporated these considerations explicitly into our revised discussion. We have also added the time taken for running RecPD on our simulated and real-world examples and foresee future improvements which will increase computational efficiency.

RecPD and derived measures do not adjust for differential abundance of entities (taxa) carrying ‘features’. As described in the text, RecPD is an adjusted richness measure, but does not account for the frequency with which each trait/feature is observed in a community. I believe this is a relevant limitation of the method that should at least be addressed in the text – also to put RecPD in context of existing measures, see previous point.

RESPONSE: The reviewer is correct that does not account for differential abundances, so care must be taken when comparing multiple features that have different abundance in the study population. We explicitly point this out in the revised discussion.

Related to this, a more formal description of RecPD would be desirable. While the text and Fig 1 lay out the concept very well, it would be good to have more exact mathematical formulations, probably in the Methods section.

RESPONSE: We have provided a more formal description of RecPD as the first section of the Methods section and an additional table describing all the measures we have devised in the text as a potentially useful reference for the reader.

Moreover, while the simulations provide some intuition of how RecPD values behave relative to Faith's PD, the interpretation (or indeed, interpretability) of absolute RecPD values remains unclear. How do these measures behave under different scenarios? Is RecPD bounded, what are extreme scenarios that would provide extreme values?

RESPONSE: This is an excellent point. We provide intuitive ways to interpret the RecPD measure in the section Results / Step 3: Calculation of RecPD and nRecPD

Does RecPD satisfy the properties of a true metric (I believe it does not), does it satisfy the doubling (or replication) principle (I also believe not)? These points should at least be addressed as potential caveats.

RESPONSE: We do not know if RecPD formally satisfies the properties of a metric so have removed that term and now generally refer to it a system of measurement.

While the authors provide all code as supplement, I strongly encourage them to formally release RecPD as an R package on a public repository to foster its adoption by the community (and further development).

RESPONSE: We appreciate the reviewer's recommendation and have generated an R package version of our RecPD Rscripts, which can be downloaded from https://github.com/cedatorma/recpd). We have updated our manuscript to reflect this.

I also strongly encourage the authors to revise their figures with a view to the use of colourblind-friendly palettes.

RESPONSE: Done. Thank you for pointing this out.

REVIEWER #2

In the manuscript "RecPD: A Recombination-Aware Measure of Phylogenetic Diversity" the authors propose a diversity metric that accounts for recombination and generally patterns of gene gain/loss. The authors essentially expand upon the useful metric of Faith's PD to infer ancestral states at nodes, and then to calculate the time in the tree of a gene gain or loss.

This is a clever paper that proposes a novel metric that has good potential to be of use to the microbial ecology and microbial genetics broadly. I appreciated the modularity of the developed method, that it can take ancestral state reconstructions derived from different methods for comparison. I also appreciated that this metric is very simple and intuitive. That is a major strength.

RESPONSE: Thank you very much for your positive comments

I strongly recommend the authors make an implementation of RecPCD that is available to the academic community, such as through an R package, for example. There are so many different diversity statistics out there. Making RecPD an accessible statistic to calculate (through a package or python module) could make the difference between this being a widely used metric, or one that is not.

RESPONSE: We have generated an R package version of our RecPD Rscripts, which can be downloaded from https://github.com/cedatorma/recpd. We have updated our manuscript to reflect this.

In general, the figure legends were not detailed enough to explain the figures. For this manuscript specifically I would recommend including the major takeaways for the figure in the legend. Otherwise it is difficult to understand to what the reader should pay attention in the simulation results.

RESPONSE: We thank the reviewer for their critical input, and have accordingly added the major takeaway points to our figure legends.

Minor comments:
Line 64: Should read "distributions of a substantial…"
Line 80: Should read "individuals that carry"
Line 168: Should read "allow for the incorporation"
Line 219: I don't see Clustering referenced in Figure S2.
Figure 4: The caption for this figure should be expanded to explain major takeaways from figure.
Line 316: Should read "has at least…"
Line 323: Should read "based on their…"
Line 334: Sentence starting with "Notably" is too long.
Lin 771: I don't understand the statement "Branches are coloured according to overlap between RecPD-inferred gene family lineages".
Line 428: Should read "In addition to quantifying…"
Line 492: Should it read "that will then be randomly mapped"?

RESPONSE: All corrections have been made.
